# Phage Inactivation of *Listeria monocytogenes* on San Daniele Dry-Cured Ham and Elimination of Biofilms from Equipment and Working Environments

**DOI:** 10.3390/microorganisms4010004

**Published:** 2016-01-05

**Authors:** Lucilla Iacumin, Marisa Manzano, Giuseppe Comi

**Affiliations:** Department of Food Science, Università degli Studi di Udine, via Sondrio 2/a, 33100 Udine, Italy; lucilla.iacumin@uniud.it (L.I.); marisa.manzano@uniud.it (M.M.)

**Keywords:** bacteriophages, dry cured ham slices, working equipment, *Listeria monocytogenes*, biofilm, reduction

## Abstract

The anti-listerial activity of generally recognized as safe (GRAS) bacteriophage Listex P100 (phage P100) was demonstrated in broths and on the surface of slices of dry-cured ham against 5 strains or serotypes (*i.e.*, Scott A, 1/2a, 1/2b, and 4b) of *Listeria monocytogenes*. In a broth model system, phage P100 at a concentration equal to or greater than 7 log PFU/mL completely inhibited 2 log CFU/cm^2^ or 3 log CFU/cm^2^ of *L. monocytogenes* growth at 30 °C. The temperature (4, 10, 20 °C) seemed to influence P100 activity; the best results were obtained at 4 °C. On dry-cured ham slices, a P100 concentration ranging from 5 to 8 log PFU/cm^2^ was required to obtain a significant reduction in *L. monocytogenes*. At 4, 10, and 20 °C, an inoculum of 8 log PFU/cm^2^ was required to completely eliminate 2 log *L. monocytogenes*/cm^2^ and to reach the absence in 25 g product according to USA food law. Conversely, it was impossible to completely eradicate *L. monocytogenes* with an inoculum of approximately of 3.0 and 4.0 log CFU/cm^2^ and with a P100 inoculum ranging from 1 to 7 log PFU/cm^2^. P100 remained stable on dry-cured ham slices over a 14-day storage period, with only a marginal loss of 0.2 log PFU/cm^2^ from an initial phage treatment of approximately 8 log PFU/cm^2^. Moreover, phage P100 eliminated free *L. monocytogenes* cells and biofilms on the machinery surfaces used for dry-cured ham production. These findings demonstrate that the GRAS bacteriophage Listex P100 at level of 8 log PFU/cm^2^ is listericidal and useful for reducing the *L. monocytogenes* concentration or eradicating the bacteria from dry-cured ham.

## 1. Introduction

*Listeria monocytogenes* is an opportunistic pathogenic microorganism that is often implicated in food-borne diseases. The resulting disease can be invasive (bacteremia and meningitis) or non-invasive (gastroenteritis accompanied by fever and vomiting) [1]. *L. monocytogenes* is a ubiquitous microorganism and can be found in many food products, such as vegetables and vegetable products, milk and dairy products, and meat and meat products [2,3,4,5,6]. *L. monocytogenes* grows in food at 1–4 °C [7,8,9,10]. However, foods with water activity (Aw) less than 0.92 and acidic pH (< 4.0) do not support its growth. The bacteria may still be present in many processed products, particularly those that do not provide thermal treatment post-packaging. The use of the Hazard Analysis Critical Control Point, Good Manufacturing Practices and Sanification Standard Organization Program systems (HACCP, GMP, and SSOP) has led to a decrease in the presence of *L. monocytogenes* in processing environments and food, but its eradication is still far from complete [11]. This issue creates concerns for the food industry and for those responsible for food safety monitoring [8,9,11,12]. EEC Reg. 2073/05 (EEC Commission 15 November 2005, Gazzetta Ufficiale L 338/1, 22 December 2005) imposed criteria for the acceptability of food regarding the presence of *L. monocytogenes*. For “ready-to-eat” products that do not support its growth, EEC Reg. 2073/05 indicates a tolerance limit of 100 CFU/g; in all other products that support its growth, the tolerance limit is indicated as “0 tolerance” (Absence in 25 g product). The concept of “0 tolerance” is applied in many countries regardless of the food type, although it is accepted that *L. monocytogenes* is a ubiquitous microorganism and it is often impossible to completely eradicate it from food and processing environments. In the USA, the law provides for the total absence of *L. monocytogenes* in meat and meat products (FDA/FSIS, [13,14]) and requires manufacturers of ready-to-eat foods to validate their anti-listeria processes.

Dry-cured ham is one of the meat products that is identified as ready-to-eat that does not support the growth of *L. monocytogenes*; consequently, in Europe it meets the criteria set by EEC Reg. 2073/05. Specifically, this regulation sets an acceptable limit of 100 *L. monocytogenes*/g. The technology used for dry-cured ham production prevents the development of any pathogen derived from raw meat or the environment [15]. However, it is possible that the microorganism remains viable on the dry-cured ham because *L. monocytogenes* can be introduced during the deboning or slicing phases. Nevertheless, ham producers have implemented severe HACCP or SSOP plans that have resulted in a large decrease in the presence of *L. monocytogenes* during all phases of the production process (from raw material to dry-cured ham). In fact, the rate of *L. monocytogenes* isolation from dry-cured hams (whole, in pieces, deboned or sliced) has been reduced to levels below 0.2%, resulting in a lower probability of the risk of listeriosis in consumers. Indeed, no cases of listeriosis have been reported in people who have eaten dry-cured ham [11]. However, the presence of *L. monocytogenes* contributes to problems in exporting products to countries where the 0 tolerance policy is applied (particularly the USA and Japan). In these countries, the combination of several control methods (*i.e.*, the Aw < 0.92 and refrigeration (FDA/FSIS-14)) is deemed sufficient to prevent the growth of *L. monocytogenes* but not to completely eliminate its presence in dry-cured ham accidentally contaminated during deboning or slicing. The food industry has tried several technologies to achieve 0 tolerance in ready-to-eat products. These include the use of heat treatments post-packaging, acids or organic salts, essential oils and alcohols; these treatments are spread on the surface of the product prior to packaging [11,16,17]. However, the effectiveness of the treatments is strictly dependent on the product, the intensity of the physical agent and the concentration of the chemical agent. The use of bacteriophages could represent a promising approach for the control of *L. monocytogenes* and other pathogens [16,18,19,20,21] on meat products. Bacteriophages are specific “viruses” of microbial cells; they are specific to the different serotypes or strains of microbial species and are obligate parasites with a genetic parasitism. Once in contact with the target cell, bacteriophages inject their DNA and force the cell to produce the bacteriophage genome and structures (e.g., capsid and tail). When the phages are complete, they lyse the cells, pour outside, and infect other cells. As a consequence, bacteriophage infection can lead to the destruction of the entire colony [18,20,22,23]. Bacteriophages are ubiquitous in nature and can be isolated from soil, water [21] and foods such as meat and meat products, dairy products and vegetables [24,25,26,27,28]. Over the last year, the FDA/USA approved a preparation of bacteriophages (LISTEX P100) to combat the presence of *L. monocytogenes* directly in foods [29,30,31].

Therefore, the purpose of our study was to investigate the efficacy of phage Listex P100 against *L. monocytogenes* intentionally inoculated onto slices of dry-cured ham. The biocontrol of *L. monocytogenes* was evaluated according to the phage dose, phage contact time, storage temperature and storage time. Its action against *L. monocytogenes* biofilms on model surfaces was also evaluated to simulate the working surfaces of dry-cured ham facilities.

## 2. Materials and Methods

### 2.1. Listeria Monocytogenes Strains and Suspensions

The inocula consisted of 5 *L. monocytogenes* strains from the International Collections and Collection of the Department of Food Science, University of Udine (DSA): *L. monocytogenes* Scott A, *L. monocytogenes* NCTC 7979 (serotype 1/2a), *L. monocytogenes* NCTC 10887 (serotype 1/2b), *L. monocytogenes* NCTC 10527 (serotype 4b), and *L. monocytogenes* DSA25. All of the strains were isolated from meat products. Each suspension was prepared with a loopful of *L. monocytogenes* added to sterile peptone water (0.8% NaCl). The suspensions were diluted to a final optical density at 650 nm (OD_650_ = 1.0). To determine the suspension concentration, serial dilutions were prepared in peptone water, and 0.1 mL of each dilution was inoculated onto brain heart infusion agar plates (BHI Agar, Oxoid, Italy). The plates were incubated at 37 °C for 48 h and then the colonies were counted. Each suspension contained an average of 10^7^ CFU/mL. Serial dilutions were used to obtain the concentrations of the inocula. Each strain was used in single or mixed inocula depending on the tests, as described below.

### 2.2. Evaluation of the Activity of the Bacteriophage P100 Listex in Broth and Agar

#### 2.2.1. Assessment of Phage Concentration (Titer)

Briefly (Soni and Nannapanemi, 23, modified): Serial dilutions of the phage suspension (P100) were prepared in a sterile buffer (100 mM NaCl, 10 mM MgSO_4_, and 50 mM Tris-HCl [pH 7.5]). Briefly, 100 μL of each dilution was mixed with 100 μL of any *L. monocytogenes* serotype or strain suspension. After mixing, both suspensions were added to 4 mL of semi-solid agar (tryptic soy broth with 0.4% agar; Oxoid, Italy). Then, the mixture was distributed into plates containing tryptic soy agar (Oxoid, Italy) and allowed to solidify. The plates were incubated at 30 °C for 18–24 h, and then the lysis plaques were counted. Three replicates were performed for each test. The experiment was done as one experiment on the same day, with three technical parallels repeated test.

#### 2.2.2. Effect of the P100 Suspension Activity against Different Concentrations of *L. monocytogenes* (2 and 3 log CFU/mL) at 30 °C in Broth

Briefly (Soni and Nannapaneni, 23 modified): The inocula consisted of a mix of different strains or serotypes of *L. monocytogenes* in suspension. The phage titers tested were approximately from 1 to 10 log PFU/mL. Brain heart infusion broth (BHI, Oxoid, Italy) was used. After inoculation of both suspensions, *L. monocytogenes* inhibition was determined in a 96-well plate. An untreated control without the phage suspension was also prepared. Three replicates were performed for each treatment on the same day. The microplates were incubated at 30 °C for 30 min to obtain temperature equilibration prior to the addition of the phage suspension. At specific intervals, the microplates were read at an OD of 630 nm on a microplate absorbance reader (Sunrise, Tecan, CH). The results were expressed as the mean detection time (DT) obtained through the detection of the inflection point of the growth curve.

#### 2.2.3. Effect of Temperature on the Activity of the P100 Suspension against *L. monocytogenes*

The *L. monocytogenes* suspension was obtained by mixing all strains or serotypes. The concentration of the phage suspension ranged from 1 to 10 log PFU/mL. The method used is described in Section 2.2.1. The plates were incubated at 4, 10, or 20 °C for 2 h. Three replicates were performed on the same day.

### 2.3. Application of P100 to Control L. monocytogenes on Dry-Cured Ham Slices

The samples included San Daniele dry-cured ham (Aw 0.90) slices. Serial dilutions of *L. monocytogenes* strain or serotype suspensions were prepared. *L. monocytogenes* strain or serotype suspensions were used individually or in a mixture and were inoculated onto slices of dry-cured ham at concentrations of 2, 3, or 4 log CFU/cm^2^ depending on the type of test. The phage suspension was diluted up to values of 4 log PFU/mL, and appropriate aliquots of each dilution were plated on the slices inoculated with *L. monocytogenes*. Briefly: 1 mL of different concentrations of *L. monocytogenes* was spread onto the surface of dry cured ham slices. After 30 min, 1 mL of different P100 suspension titers was also spread on the same surface of the dry cured ham slices. The inoculated slices were packaged under vacuum in Ecoterm VP 300 film (vacuum). After the time of each experiment, different aliquots of the slices were sampled and diluted in stomacher bag. After stomaching and serial dilution, the surviving *L. monocytogenes* were counted by ISO (ISO (11290-1,2:1996 Adm.1:2004. Microbiology of food and animal feeding stuffs—Horizontal method for the detection of *Listeria monocytogenes*) method (Detection limit 10 CFU/cm^2^ or g). The enrichment procedure was also used according to the same ISO method (Detection limit < 1 in 25 cm^2^ or g).

#### 2.3.1. Control of the *L. monocytogenes* Suspension Mixture on Dry-Cured Ham Slices at Different Temperatures

The method used is described in Section 2.3. Briefly, the titers of the phage suspension used in this experiment were 8 log PFU/cm^2^. The concentrations of the *L. monocytogenes* strain mixtures were approximately 2, 3, or 4 log CFU/cm^2^. The temperatures tested were 4, 10, or 20 °C, and the samples were incubated for 24 h. Three replicates were performed for each experiment on the same day.

#### 2.3.2. Control of *L. monocytogenes* Suspension Mixtures on Dry-Cured Ham Slices with Different Phage Concentrations

The method used is described in Section 2.3. Briefly, the phage suspension titer was 8, 7, 6, 5, or 4 log PFU/cm^2^. *L. monocytogenes* (mixed suspension) was applied at concentrations of approximately 2 and 4 log CFU/cm^2^ for 24 h at 4 °C. Three replicates were performed for each experiment on the same day.

#### 2.3.3. Control of Each *L. monocytogenes* Strain Suspension on Dry-Cured Ham Slices (Aw 0.90)

The method used is described in Section 2.3. Briefly, the phage suspension titer was 8 log PFU/cm^2^. Each *L. monocytogenes* strain was applied at a concentration of approximately 2 log CFU/cm^2.^. A control made with the mix of five of *L. monocytogenes* strains but without the P100 suspension was also generated. Both the phage and *L. monocytogenes* concentrations were inoculated onto dry cured ham slices, which were packaged and incubated for 24 h at 4 °C. Three replicates were performed for each strain and control on the same day.

#### 2.3.4. Stability of the P100 Suspension *vs. L. monocytogenes* on Dry-Cured Ham Slices at 4 °C for 0, 7, or 14 Days

The method used is described in Section 2.3. Briefly, the phage suspension titers were 8 log PFU/cm^2^. *L. monocytogenes* (mixed suspension) was applied at a concentration of approximately 2 log CFU/cm^2^. After inoculation of the *L. monocytogenes* mixture and the phage suspension, the slices were packaged under a vacuum and stored at 4 °C. Nine samples were produced. At 0, 7, and 14 days, three samples were collected and washed with peptone water to recover the phages. After dilution, the phage suspension was titrated as previously described in 2.2.1.

### 2.4. Control of L. monocytogenes Biofilms by Phage Suspensions on Stainless Steel Wafers to Simulate Inert Substrates in Contact with Food in Production Facilities

The steel wafers were placed in Petri plates containing BHI broth inoculated with the mixture of the different strains or serotypes of *L. monocytogenes*. Six plates were prepared. After incubation at 30 °C for 4 days, the plates were removed and dried. Then, they were divided into two groups: three plates were not treated with the phage suspension, and the remaining three plates were treated with 8 log PFU/cm^2^ of the phage suspension. The wafers were placed in empty sterile Petri plates and incubated at 20 °C for 24 h. After this period, the treated and untreated wafers were pressed on Palcam Agar plates (Oxoid, Italy). The lack of growth in the contact area of the plates was used to assess the activity of the phage.

### 2.5. Analysis of Variance

Analysis of variance (ANOVA) was used to compare the results of the different experiments. ANOVA was calculated with the averages and standard deviations, and significant differences (*p* < 0.05) were determined by the Honest Significant Difference test (HSD test) and Tukey’s test using the StatGraphics software package from Statistical Graphics (Rockville, MD, USA).

## 3. Results

Table 1 shows the Phage 100 (P100) titer expressed in log PFU/mL assessed against different *L. monocytogenes* strains or serotypes. The total average value is equal to 10.1 ± 0.2 log PFU/mL. The values are slightly lower than those verified by Soni and Nannapaneni [23], who found Phage 100 values of approximately 11 log PFU/mL. The titer seemed to vary at the level of the strain or serotype considered. The phage titers *vs. L. monocytogenes* (Scott A, NCTC7979, and NCTC 10527) were significantly different (*p* < 0.05) from those *vs. L. monocytogenes* NCTC 10887 and DSA 25. The averages of these strains were less than 10 log PFU/mL. Conversely, the remaining strains showed a titer higher than 10 log PFU/mL. Thus, a lower resistance of these strains to the phage can be assumed.

**Table 1:** Titer of P100 suspension *vs.* different *L. monocytogenes* strains or serotypes *in vitro*.

*L. monocytogenes* Strains/Serotype	Log PFU/mL	S.D.
*L. monocytogenes* Scott A	10.6	0.5 ^a^
*L. monocytogenes* NCTC 7979 1/2a	10.7	0.3 ^a^
*L. monocytogenes* NCTC 10527 1/2b	10.2	0.3 ^a^
*L. monocytogenes* NCTC 10887 4b	9.5	0.2 ^b^
*L. monocytogenes* DSA 25	9.7	0.1 ^b^

Legend: Data represent the means (Log PFU/mL) ± standard deviations (S.D.) of the total samples; Mean with the same letters within the same column (following the values) are not significantly different (*p* < 0.05).

The effect of the P100 titer was evaluated against different concentrations of the *L. monocytogenes* mixtures (2 and 3 log CFU/mL) at 30 °C in broth (Table 2). The results were expressed in DT (detection time), which represents the value of the inflection point of the growth curve. Complete *L. monocytogenes* inhibition was observed when the P100 titer was greater than or equal to 7 log PFU/mL. The lethal effect was independent of the initial *L. monocytogenes* concentration (either 2 log CFU/mL or 3 log CFU/mL). P100 titers less than 6 log PFU/mL did not permit the complete elimination of *L. monocytogenes*, and the DT decreased with the decreasing P100 titer. P100 concentrations less than 6 log PFU/mL showed an inhibitory action that was strictly dependent on the concentration of the phage. The DT of *L. monocytogenes* at a concentration of 2 log CFU/mL was higher than the DT at 3 log CFU/mL. No significant differences were found among the DTs when the P100 inoculum concentration was between 1 and 6 log PFU/mL and the *L monocytogenes* concentration was 3 log CFU/mL (*p* > 0.05). Conversely, significant differences were observed when the *L. monocytogenes* inoculum concentration was equal to 2 log CFU/mL and the P100 titer was between 1 and 6 log PFU/mL (*p* < 0.05).

The stability of the P100 suspension (10 log PFU/mL) at 4, 10 and 20 °C was evaluated *in vitro* against a mix of *L. monocytogenes* strains ((2 log CFU/mL). The temperatures tested did not influence the infectivity or the stability of P100 (*p* > 0.05).

*L. monocytogenes* reduction as a function of the temperature and inoculum concentration is highlighted in Table 3. Mixtures of *L. monocytogenes* strains were inoculated onto dry-cured ham slices at concentrations of 2, 3, and 4 log CFU/cm^2^ and exposed to P100 at a titer at 8 log PFU/cm^2^. To detect *L. monocytogenes*, when its inocula were at level of 2 and 3 log CFU/cm^2^, the enrichment method had to be used. *L. monocytogenes* was completely inactivated regardless of the storage temperature, when its initial inoculum was equal to 2 log CFU/cm^2^. Conversely, when its inocolum was 3 log CFU/cm^2^, it was present on the 25 cm^2^ surface. Finally, when the initial *L. monocytogenes* inocolum was at level of 4 log CFU/cm^2^, the values of the survived *L. monocytogenes* increased with the increasing of the temperature. Therefore, it could be concluded that the reduction seemed to be influenced by the temperature and by the high concentration of the *L. monocytogenes* inoculum (4 log CFU/cm^2^). In this case, the reduction was increased at 4 °C (*p* < 0.05).

**Table 2:** Effect of P100 titer activity *vs. L. monocytogenes* mix strains suspension at 30 °C in BHI.

P100 Titer	*L. monocytogenes* Concentration	
	2 log CFU/mL	3 log CFU/mL
PFU/mL	DT (h)	DT (h)
10^10^	∞ ^a^	∞ ^a^
10^9^	∞ _a_	∞ ^a^
10^8^	∞ ^a^	∞ ^a^
10^7^	∞ ^a^	∞ ^a^
10^6^	18.00 ± 0.30 ^b^	13.00 ± 1.00 ^b^
10^5^	17.30 ± 1.00 ^b^	13.00 ± 1.00 ^b^
10^4^	13.30 ± 2.00 ^c^	11.30 ± 2.00 ^b^
10^3^	13.30 ± 1.00 ^c^	11.00 ± 2.00 ^b^
10^2^	13.00 ± 1.00 ^c^	11.00 ± 1.00 ^b^
10^1^	12.30 ± 0.30 ^c^	12.00 ± 0.30 ^b^
10^0^	12.30 ± 0.30 ^c^	12.00 ± 1.00 ^b^
Control	12.00 ± 1.00 ^c^	12.00 ± 0.30 ^b^

Legend: D.T.: Detection time; ∞: No growth; Control: No P100 inocolum. Data represent the means ± standard deviations of the total samples; Mean with the same letters within the same column (following the values) are not significantly differently (*p* < 0.05).

**Table 3:** Survival of a mix of 5 *L. monocytogenes* strains on dry cured ham slices at different temperatures and inocolum concentrations after exposure to P100 phage.

Temperature	2 log CFU/cm^2^	3 log CFU/cm^2^	4 log CFU/cm^2^
4 °C	Not detected/25 cm^2^	Detected/25 cm^2^	0.3 log ± 0.1 ^a^
10 °C	Not detected/25 cm^2^	Detected/25 cm^2^	2.2 log ± 0.2 ^b^
20 °C	Not detected/25 cm^2^	Detected/25 cm^2^	3.0 log ± 0.1 ^c^

Legend: mix of *L. monocytogenes*: 2-3-4 log CFU/cm^2^—P100 8 log PFU/cm^2^: Data represent the means (Log CFU/cm^2^) ± standard deviations (S.D.); Mean with the same letters within the same column (following the values) are not significantly different (*p* < 0.05).

The US Food and Drug Administration approved the use of Listex P100 to reduce the concentration of *L. monocytogenes* in food (fresh and ready-to-eat food) only when a titer up to 8 log PFU/g was used. Therefore, It was considered appropriate to investigate the use of P100 *in situ.* P100 with a titer up to 8 log PFU/cm^2^ was used against different *L. monocytogenes* strains on dry-cured ham slices.

Table 4 shows the effect of the phage concentration on the reduction of *L. monocytogene*s inoculated onto dry-cured ham slices. The increase in the phage titer led to different decreases in the *L. monocytogenes* concentration. A phage titer of 8 log PFU/cm^2^ completely reduced the *L. monocytogenes*, when its concentration was about 2 log CFU/cm^2^. It was not detected also by enrichment colture (Detection limit < 1 CFU/25 cm^2^). However, *L. monocytogenes* was still present in the 25 cm^2^ area, when the P100 titer was from 5 to 7 log PFU/cm^2^. A minor decrease was observed using the same phage titer against a concentration of *L. monocytogenes* equal to 4 log CFU/cm^2^. In this case, only phage treatment with 8 log PFU/cm^2^ permitted the surviving of a concentration of about 1.5 log CFU/cm^2^ of *L. monocytogenes*. This reduction was significantly different (*p* < 0.05) from that observed with the other phage titers (from 7 to 4 PFU/cm^2^). The reduction values calculated using from 4 to 7 PFU/cm^2^ phage titers were not significantly different (*p* > 0.05). Based on these results, it was confirmed the importance of using a P100 titer equal to 8 PFU/cm^2^ to ensure the elimination or eradication of high concentrations of *L. monocytogenes*.

**Table 4:** Survival of a mix of 5 *L. monocytogenes* strains on dry cured ham slices at different inocolum concentrations after exposure to different P100 phage titers.

P100		*L. monocytogenes*	
Log PFU/cm^2^	2 Log CFU/cm^2^	2 Log CFU/cm^2^	4 Log CFU/cm^2^
8	Not detected	Not detected/25 cm^2^	1.5 ± 0.1 ^a^
7	Not detected	Detected/25 cm^2^	3.3 ± 0.2 ^b^
6	Not detected	Detected/25 cm^2^	3.6 ± 0.3 ^b^
5	Not detected	Detected/25 cm^2^	3.8 ± 0.1 ^b^
4	0.5 ± 0.1	2.0 ± 0.3 *	3.9 ± 0.2 ^b^
Control	2.0 ± 0.1	2.0 ± 0.1 *	4.0 ± 0.1 ^b,c^

Legend: Data represent the means (Log CFU/cm^2^) ± standard deviations (S.D.) of the survived *L. monocytogenes*; * log CFU/25 cm^2^. Mean with the same letters within the same column (following the values) are not significantly differently (*p* < 0.05).

All the strains in Table 1 were tested to value their survival against P100 phage at level of 8 PFU/cm^2^. Each *L. monocytogenes* strains was inoculated separately at concentration of 2 log CFU/cm^2^ onto dry cured ham slices and analyzed to detect the surviving cells after 24 h at 4 °C. This is the temperature used to store all of the different dry-cured ham typologies (whole, deboned, slices, and pieces). All of the *L. monocytogenes* strains exhibited similar behavior. It was observed a reduction of approximately 2 log CFU/cm^2^, and the presence of *L. monocytogenes* was not observed in the enrichment culture. These data are important confirmation of the efficiency of P100 against different *L. monocytogenes* types and serotypes. Indeed they also confirmed the data from a previous study that tested inoculation of *L. monocytogenes* mixed strains (data not shown).

The P100 stability was evaluated on dry-cured ham slices stored at 4 °C for 0, 7, and 14 days. The P100 titer was relatively stable on slices throughout the storage period. Indeed, there were no significant differences among the P100 titers at any of the investigated time points (*p* > 0.05). Consequently, these data demonstrated that the P100 activity remained stable over time.

**Figure 1:**
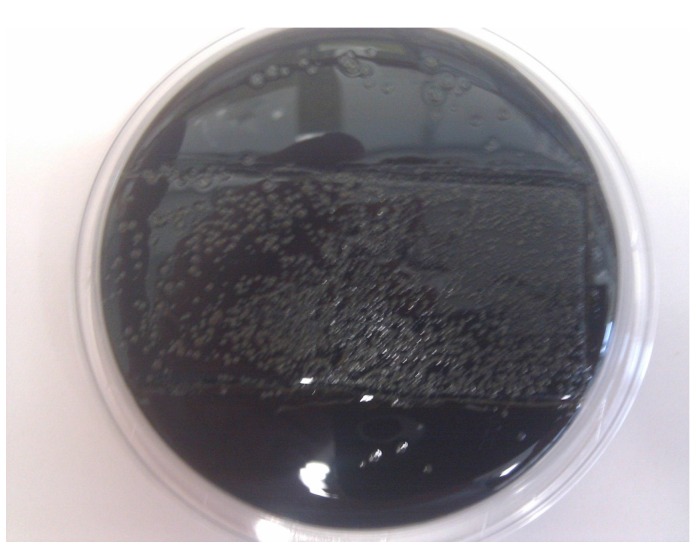
Track on Palcam agar of wafer with *L. monocytogenes* biofilm not treated with P100 phage (*L. monocytogenes* grew, hydrolyzed esculin and produced colonies surrounded by black haloes on the Palcam agar).

**Figure 2:**
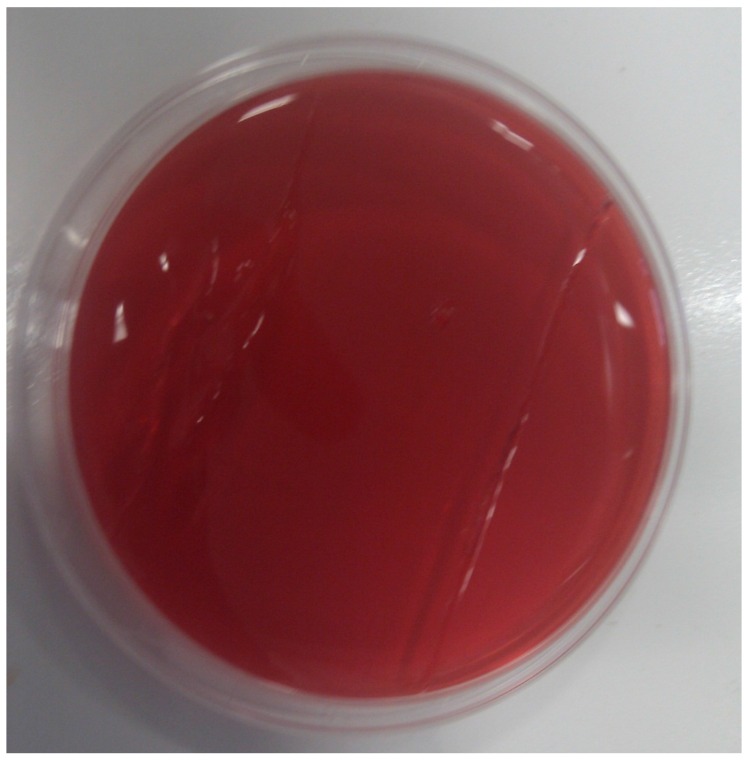
Track on Palcam agar of wafer with *L. monocytogenes* biofilm treated P100 phage (*L. monocytogenes* was eliminated by P100 phage, consequently did not grow and hydrolyze esculin and the color of the Palcam agar remained red).

Finally, it was evaluated whether P100 could eliminate *L. monocytogenes* biofilms on a stainless steel wafer, which was used to simulate an inert substrate in contact with the food. P100 treatment allowed complete biofilm elimination, as shown in Figure 1 and Figure 2. The untreated wafers resulted in the transfer of the biofilm to the Palcam agar contact plate (Figure 1), whereas wafers with a biofilm treated with 8 log PFU/cm^2^ of P100 did not transfer and no growth was observed in the Palcam agar (Figure 2).

## 4. Discussion

This study showed that the bacteriophage P100 could reduce the concentration of *L. monocytogenes* in both nutrient broth and on the surfaces of intentionally contaminated dry-cured hams. The activity was dependent on the P100 concentration, the *L. monocytogenes* inoculum concentration and the temperature. In broth, P100 was effective at concentrations between 10 and 7 log PFU/mL. Under this condition, no growth of *L. monocytogenes* was observed regardless of the bacterial concentration. Conversely, lower P100 concentrations (1–6 log PFU/mL) only reduced *L. monocytogenes* growth. The data were expressed in DT, which represented the time it took the microorganism to reach the exponential growth phase. The P100 activity on *L. monocytogenes* added to dry-cured ham was strongly dependent on the temperature and concentration. A higher concentration of phages (>8 log PFU/cm^2^) corresponded to a greater reduction of *L. monocytogenes*. The reduction was similar to that reported by other authors. Soni and Nannapanesi [23] observed that a phage suspension greater than 8 log PFU/g decreased the concentration of *L. monocytogenes* on salmon fillets by three logs. Similar results were demonstrated by Guenther *et al.* [32]. These authors found a major increase in the phage efficacy in broths compared to foodstuffs, such as ready-to-eat foods, and attributed this effect to greater phage diffusion in liquids. Indeed, different authors [16,33,34,35] obtained significant *Salmonella* and *Campylobacter* reductions with phage titers equal to 6–8 log PFU/g or cm^2^.

Bacteriophages were used for the biocontrol *L. monocytogenes* on soft ripened white mold and red-smear cheeses [36]. *L. monocytogenes* counts were decreased by more than 3 logs on red-smear cheeses ripened for 22 days, and the bacterial counts were decreased by 2.5 logs at the end of the 21 day ripening period of camembert-type cheese until the viable counts dropped below the limit of detection; in these experiments, the initial *L. monocytogenes* concentration was less than 100 CFU/g [36]. Silva *et al.*, [37] obtained similar results in soft cheese using bacteriophage P100, where the *L. monocytogenes* reduction was between 0.8 and 2.5 log units. The authors confirmed that the effectiveness of the phage treatment depended on the initial *L. monocytogenes* concentration [37].

The tests on dry-cured ham slices were performed at 4 °C for two reasons. The first is related to the intrinsic phage properties: the phages adhere to host cells more effectively at refrigeration temperatures than at room temperature [20]. The second reason is that their activity is required at 4 °C, which is the classic temperature used for fresh or processed meat storage. Consequently, it is appropriate to select phages that have a maximum adsorption activity at this temperature because the adsorption affects the reduction of the host concentration [20,38]. Moreover, the P100 suspension effectively inhibited different concentrations of *L. monocytogenes* inoculated onto dry-cured ham slices stored at 4, 10 and 20 °C. The highest inhibition was found in the presence of 2 log CFU/cm^2^·*L. monocytogenes*, which is a typical concentration due to accidental contamination [9,18]. In this case, the use of a P100 titer equal to 8 log PFU/cm^2^ allowed us to achieve 0 tolerance as required by the FSIS [13,14] at all the temperatures tested. Unfortunately, when the *L. monocytogenes* inocula were at level of 3 log CFU/cm^2^ or 4 log CFU/cm^2^, it was not possible to reach the 0 tolerance. In particular, using a *L. monocytogenes* inoculum of about 4 log CFU/cm^2^, at 4 °C the reduction was nearly 4 log CFU/cm^2^; the reduction at 10 °C was still appreciable (~1.8 log CFU/cm^2^) and at 20 °C was equal to 1 log CFU/cm^2^. The data were confirmed against all strains and serotypes investigated. Indeed, the parameters used (inoculum of 2 log *L. monocytogenes*/cm^2^ and 8 log PFU/cm^2^ of the P100 suspension and a reaction time of 24 h at 4 °C) reduced the bacterial concentration to almost 2 log CFU/cm^2^ and consequently reached the 0 tolerance level requested by the FSIS. As shown, all of the *L. monocytogenes* strains investigated were absent in the 25 cm^2^ area.

The use of the P100 suspension also inactivated *L. monocytogenes* on inert substrates (stainless steel used in the food industry). This reduction could help eradicate this microorganism from processing environments. Raw materials and processed products often undergo accidental contamination during the production phase due to the ability of *L. monocytogenes* to produce either free cells or biofilms [39].

The P100 suspension may be successfully used in the dry-cured ham production process during ripening and especially during deboning or slicing. P100 could be used before packaging to favor its antimicrobial activity. Either the deboned dry-cured ham or the slices are packaged under a vacuum or in MAP (Modified Atmosphere Packaging) and stored at 4 °C. As observed in this study, this temperature increases the efficacy of P100 *vs. L. monocytogenes* and phage virulence is maintained over time.

Dry-cured ham can often become contaminated with *L. monocytogenes*. The contamination level is usually low and is limited to 10 to 100 CFU/g or cm^2^ [11,15]; this level remains constant during the production phases because the Aw of the product is low (less than 0.92). Nevertheless, the bacteria can persist up to and beyond the deboning and/or slicing stages of dry-cured ham. Indeed, it is possible that the dry-cured ham can be further contaminated by *L. monocytogenes* present in the environment or on the equipment used during these stages. In each case, the initial contamination rarely exceeds 100 CFU/g or cm^2^. Therefore, the effectiveness of the anti-listerial agents is usually assessed against intentional inoculation of 1–2 log CFU/g or cm^2^ [7]. This concentration was also used in our experiments. Phage titers of 8 log PFU/cm^2^ demonstrated high efficacy against *L. monocytogenes* concentrations that are usually defined as accidental contamination (2 log CFU/g or cm^2^). The P100 titer reached a phage particle/bacteria ratio (phage P100/*L. monocytogenes*) equal to 10^6^/1, 10^5^/1 and 10^4^/1, whereas *L. monocytogenes* was inoculated at a concentration of 2, 3 and 4 log CFU/cm^2^. In dry-cured ham, these ratios resulted in complete *L. monocytogenes* elimination, as demonstrated by other authors in various foods [23,40]. For instance, Carlton *et al.* [41] obtained a significant reduction of at least 3.5 log units or a complete eradication of *Listeria* viable counts in contaminated cheese. In agreement with our data, they found that the effect depended on the duration, frequency and dosage of phage applications. Moreover, the phage/bacteria ratio was clearly favorable to the phage because higher phage concentrations represented an increased probability that the phage would contact the target cells [19].

Therefore, the phage concentration used could reduce the inoculated *L. monocytogenes* concentration or eliminate it completely. This elimination was observed at 4 °C within 24 h. Using predictive models, Bigwood *et al.* [34] also showed that significant decreases could be observed within 1 h. According to our data, this short time frame could not be used because the aim was to achieve not only a significant reduction but also to reach 0 tolerance. Achieving the 0 tolerance level is important for products contaminated with *L. monocytogenes* bound for export in the USA, Japan and now China; these countries require the absence of *L. monocytogenes* in 25 g of food products. The use of a long time period for “phage activity” was necessary because the phage fully exploited its potential over time. Therefore, it was assessed the phage stability on dry-cured ham during storage at 4 °C for 14 days. The data confirmed an excellent stability, even though the product had a low Aw (0.90). Phages are more stable in media with an Aw content > 0.97 [19,32]. The phage concentration was not sufficient to ensure the contact of the viral particles with the target, which was more important than the food typology. The latter factor must allow the passive diffusion of the phage so that it can reach the target cells and kill them. In liquid food (milk and cheese in brine), the possibility of contact does not seem to be problematic because the phage can diffuse almost freely [32]. The phage behavior can be different for solid foods, foods with a smooth surface (hot dogs and salads) or foods with an irregular surface (seafood and fresh and processed meat). Solid foods are more difficult to treat than liquids because the distribution of phage particles is physically limited. The phages are not inactivated by these foods [19,23,41,42,43,44]. Therefore, the limited diffusion and reduced contact of phage particles with the bacteria can be assumed to be the main cause of the decrease in the efficiency. This obstacle can be overcome by modifying the phage application methods (*i.e.*, with the use of a higher phage concentrations, large liquid volumes and/or phage application prior to the initiation of the microbial contamination [32,42]. It should also be stressed that it is necessary to use a sufficiently high phage concentration from the beginning. However, the phage concentration increases because phages grow in the target cells. The number of new particles is estimated to be 40–50 for each infected cell.

## 5. Conclusions

This study showed a high listericidal activity of the P100 (bacteriophage P100 Listex) on dry-cured ham against 5 different strains or serotypes of *L. monocytogenes*. Because the phage is recognized by GRAS [31], its use could easily replace other invasive technologies that leave residues such as chlorine dioxide, ozone or irradiation. Phage employment may be accepted by consumers because they always search for “natural” foods subjected to less aggressive treatments that are free of chemical preservatives [37,44]. Bacteriophages, which are agents that infect only bacteria without changing the food color, odor and flavor, could represent an adequate and safe way to control pathogens in foods [44].
